# Autonomous Deployment of Underwater Acoustic Monitoring Devices Using an Unmanned Aerial Vehicle: The Flying Hydrophone

**DOI:** 10.3390/s20216064

**Published:** 2020-10-25

**Authors:** Daniel Babatunde, Simon Pomeroy, Paul Lepper, Ben Clark, Rebecca Walker

**Affiliations:** 1 Wolfson School of Mechanical, Electrical and Manufacturing Engineering, Loughborough University, Loughborough LE11 3TU, UK; s.c.pomeroy@lboro.ac.uk (S.P.); p.a.lepper@lboro.ac.uk (P.L.); B.Clark@lboro.ac.uk (B.C.); 2 Natural England, 2nd Floor, Dragonfly House, 2 Gilders Way, Norwich NR3 1UB, UK; Rebecca.Walker2@naturalengland.org.uk

**Keywords:** unmanned aerial vehicles, autonomous vehicles, passive acoustic monitoring, harbour porpoises, underwater acoustic devices

## Abstract

Unmanned aerial vehicles (UAV) are increasingly becoming a popular tool in the observation and study of marine mammals. However, the potential capabilities of these vehicles regarding autonomous operations are not being fully exploited for passive underwater acoustic monitoring in marine mammal research. This article presents results from the development of a UAV system equipped with an underwater acoustic recorder aimed at assisting with the monitoring of harbour porpoises in Special Areas of Conservation in the United Kingdom. The UAV is capable of autonomous navigation, persistent landing, take-off and automatic data acquisition at specified waypoints. The system architecture that enables autonomous UAV flight including waypoint planning and control is described. A bespoke lightweight underwater acoustic recorder (named the PorpDAQ) capable of transmitting the results of fast Fourier transforms (FFT) applied to incoming signals from a hydrophone was also designed. The system’s operation is successfully validated with a combination of outdoor experiments and indoor simulations demonstrating different UAVs capable of autonomously navigating and landing at specific waypoints while recording data in an indoor tank. Results from the recorder suggest that lightweight, relatively low-cost systems can be used in place of heavier more expensive alternatives.

## 1. Introduction

An increasing demand for natural resources from human activities has driven the expansion of interaction with the earth’s oceans. In certain sectors, the reliance on fossil fuels for producing energy is reducing due to the increased use of alternative sources of energy such as wind and solar. For example, in 2019, the UK National Grid produced more electricity from zero carbon sources compared to fossil fuels with zero carbon sources accounting for 47.9% of energy produced [1]. From these zero carbon sources, wind accounted for 19% of the total energy produced [1] with offshore wind accounting for 9.9% [2]. Additionally, almost 10 gigawatts of offshore wind energy has been installed as of 2019 with a target of 40 gigawatts by 2030 as part of the government’s commitment of net zero emissions by 2050 [3]. However, the increase in lower carbon energy sources comes with an increase in anthropogenic activity in the oceans that has the potential to negatively impact marine ecosystems. One area of growing concern is in the introduction of human-made underwater acoustic noise due to increased offshore activity. Activities such as seismic surveys to find oil and gas reserves and pile driving to install offshore wind foundations generate large amounts of sound energy underwater which potentially alter the behaviour and cause injury and permanent damage to marine mammals [4]. The effect of these activities on marine life has become a cause of concern to relevant environmental authorities for the protection and conservation of marine mammals as well as to licensing and governing bodies across almost all marine industries. An example of this is the introduction of the Marine Strategy Framework Directive (MSFD) [5] which was introduced in 2008 in order to improve the protection of marine environments across Europe and the UK Marine Strategy which sets out the framework for implementing the MSFD in UK waters [6]. The directive requires member states of the European Union to develop strategies to achieve Good Environmental Status (GES) in marine waters by 2020. The concern towards the increasing amount of anthropogenic underwater noise is shown in Descriptor 11 [7,8,9] which states that the energy introduced to a marine environment (underwater noise included) should be at levels which do not have a negative effect on the ecosystem. Descriptor 11 also publishes guidance for member states to monitor underwater noise in their respective waters. Another example can be found in the mitigation guidelines for minimising the risk of injury to marine mammals when carrying out offshore seismic surveys published by the Joint Nature Conservation Committee (JNCC) [10] and other interested parties [11,12]. As a result of this, many offshore surveys for oil and gas exploration require the use of Marine Mammal Observers (MMOs) and Passive Acoustic Monitoring operators to monitor and mitigate potential impacts of noise on marine wildlife. Some marine species and habitats are also provided additional protection through the designation of Special Areas of Conservation (SACs).

About 25% of UK waters are designated as Marine Protected Areas with 309 inshore and 73 offshore sites. Ninety-one of these sites are designated as Marine Conservation Zones (MCZs) while 115 (14%) are designated as SACs [13]. One marine mammal species with designated protection status is the harbour porpoise (*Phocoena phocoena*). Harbour porpoises are the most common cetacean found in UK waters with a reported population of 197,579 [14]. As of January 2019, seven SACs have been designated for harbour porpoise in UK waters; the Bristol Channel Approaches, West Wales Marine, North Anglesey Marine, North Channel, the Southern North Sea the Inner Hebrides and Minches and the Skerries and Causeway [15]. The current environmental status of the harbour porpoise as set out in descriptors 1 and 4 of the UK Marine Strategy is “unknown” [13]. The harbour porpoise has also been assigned a “least concern” classification on the International Union for Conservation of Nature (IUCN) Red List of threatened species [16].

Monitoring the conservation status of marine mammals requires various assessments. Some of the techniques involved include assessment of their distributional pattern, abundance estimation to determine their numbers in a specific area [17], estimating the changes in population [18] and tracking seasonal variations in a geographical area [19,20]. Abundance and distribution can be determined using several methods with visual and acoustic techniques being the most common [21,22]. Visual observations of harbour porpoises are used to collect information on their presence and surface behaviour and are commonly carried out from planes [23,24], ships [18,25,26,27], and in some cases, from land [28]. Acoustic-based surveys are dependent on the underwater vocalisations of the species of interest and can be achieved through passive acoustic monitoring. This involves the mooring or deployment of hydrophones either in a static position to assess variations of bottlenose dolphins and harbour porpoises in a geographical area [29,30], as drifters in tidal areas [22,31] or by being towed using a vessel to detect beaked whale echolocation clicks in real-time [32] and tracking the position of harbour porpoises in tidal rapids. Passive acoustic monitoring is also often used in combination with visual observations in survey of marine mammals and has been used to in beaked whale and harbour porpoise studies [32,33]. Other less common methods of monitoring harbour porpoises include using satellite transmitters to track tagged individuals to determine their distribution and habitat use [34].

Currently, there is a requirement to develop new monitoring techniques to detect changes in the distribution and abundance of harbour porpoises and other cetaceans in near real-time [16]. As limited area-based conservation is not enough to monitor the status of the harbour porpoises due to their mobile nature, it is crucial to develop tools that can be quickly deployed with as wide a range as possible over the habitat of the species. While the negative effect of anthropogenic activities on marine mammals in the oceans is reasonably documented, the lack of frequent data on distribution and abundance on a large scale makes it difficult to determine the effects of man-made activities on harbour porpoise’s abundance and distribution [35]. This issue also extends to the assessment of the effectiveness of mitigation measures to improve the conservation of cetaceans in specific areas [30]. One of the reasons for this is primarily because current monitoring methods are expensive, time-consuming and require a lot of resources to set up and analyse the data. While large-scale surveys provide estimates of cetaceans over a broader scale and cover all UK waters, the costs associated with them hamper the ability for observers to estimate abundance and distribution on a regular basis. This inevitably creates data gaps between the intervals of the surveys which hinders the ability to detect changes in the species’ status. Consequently, less expensive monitoring techniques through the use of passive acoustic monitoring that provide fine-scale temporal and spatial data on the presence of marine mammals in specific areas are required to assist relevant authorities with the reduction of anthropogenic impacts on cetacean species. There is an abundance of these devices available ranging from off-the-shelf devices [36,37,38,39] which are light enough to be attached as a payload for small multirotor systems to bespoke in-house systems used for specific use-cases [40,41]. These devices can be moored to collect useful information on a species over a long period [29] or can be deployed as drifters in tidal areas [22,31]. Although these systems offer the advantage of long term monitoring at relatively low costs compared to boat-based surveys and can also provide useful data on long and short term trends, there is a risk of missing relevant data due to the issues surrounding deployment and the delays associated with retrieving the data present on these systems. Additionally, some of these systems are either too cost-prohibitive mostly due to the fidelity of the data they produce or do not have the capabilities of real-time data acquisition and on-board storage. In cases where the acoustic recorder is statically deployed on the seafloor, there are additional costs attached with the maintenance and retrieval of data present on the systems.

In order to address the need for new monitoring techniques to augment the capabilities of existing methods, a system was developed to enable passive acoustic monitoring through the use of multirotor UAVs. Recently, unmanned aerial vehicles have proven to be useful tools for marine mammal researchers. They are relatively inexpensive compared to other monitoring techniques described above and they can be configured for a variety of uses. The most popular use of UAVs in marine mammal research have mostly been through visual observation of marine species using both fixed-wing and multirotor vehicles [42,43,44]. These vehicles usually have a camera attached to them, which enables observers to analyse images which are either stored in memory or relayed in real-time. While there is growing interest in attaching acoustic monitoring equipment to record underwater sound [45] and monitor marine mammals [46], there is still an existing application gap in the use of unmanned aerial vehicles for underwater acoustic monitoring through the application of robotics and automation.

This article presents current progress towards developing an autonomous unmanned aerial vehicle system for monitoring the presence and vocalisations of cetacean species, specifically the harbour porpoise. The vision for the system is one which can offer some of the capabilities of large-scale surveys by having a UAV with a camera attached for visual observation while also being capable of providing some of the capabilities of the acoustic recorders used for passive acoustic monitoring. The system could also find some application in real-time mitigation for known noisy activities where the use of marine mammal observation and passive acoustic monitoring is readily present. This development is split into two parts; the development of software to enable autonomous waypoint navigation and recording on a UAV and the design of a data acquisition recorder which will be installed on the UAV. While the system will eventually be deployed on a waterproof platform, it is important to note that at this current stage of development, there are no waterproofing capabilities present on the platform. Section 2 describes the system architecture of both the aerial vehicle and the data acquisition system. The software architecture is presented in Section 3. Results from both simulated and real experiments are included in Section 4 and finally the limitations and conclusion is presented in Section 5.

## 2. Hardware Architecture

The system described is designed with the intention to be deployed at the SACs mentioned in Section 1 and is primarily made of two subsystems:

1. A UAV capable of autonomous navigation and persistent landing and take-off on water. The system described will enable a UAV to fly to predefined GPS waypoints using a high-level interface designed on a mobile device. Using an autonomous UAV system has the advantage of introducing an agile, more manoeuvrable method for rapid deployment of marine mammal monitoring equipment. UAVs also potentially reduce the cost of marine mammal surveys and enable faster deployment and data acquisition for short range operations. Using UAVs in this context will help reduce the data gaps between large survey intervals. There is also the potential to reduce human error and save lives as autonomous systems completing repetitive tasks reduce the risk to human lives, especially far out at sea.
2. A low cost data acquisition system that records and captures underwater acoustic signals between 90 and 150 kHz (3 dB). The system is an inexpensive and light weight alternative to the current options available in the market. Designed using mostly off-the-shelf parts and weighing less than 250 g without the hydrophone element, it is integrated with the UAV and records for user-defined periods when the UAV lands on water.

### 2.1. UAV

**Figure 1:**
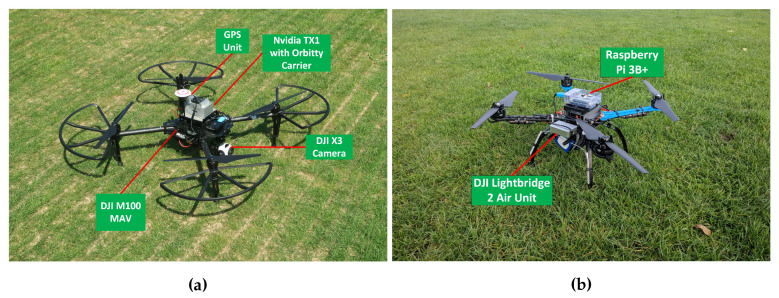
The UAV platforms; DJI M100 (**a**) and the custom quadcopter (**b**).

Two different multirotor UAVs were used for this experiment. The first is the DJI Matrice 100 (M100) as shown in Figure 1a. It has a maximum flight time of 40 min with a maximum take-off weight of 3.6 kg. The M100 is equipped with the N1 Flight controller which provides attitude control and telemetry accessible through the Onboard SDK [47] at up to 100 Hz through a UART interface. The second multirotor UAV is (Figure 1b) is a custom quadcopter with a 500 mm wheelbase equipped with the DJI N3 flight controller and DJI lightbridge to enable attitude and telemetry control.

The UAV platforms are equipped with single-board computer (SBC) options. The small form factor, low power consumption and relatively high compute power meets the UAV’s payload requirements. The Nvidia Jetson TX1 compute module with a carrier board is installed on the M100 while a Raspberry Pi 3B+ is installed on the custom UAV. The TX1 is powered by a separate 3S Li-Po battery with a total weight of 400 g while the Raspberry Pi is powered by a 5000 mAh powerbank for a total weight of 250 g. The Robot Operating System framework (ROS) together with the DJI Onboard SDK (https://github.com/dji-sdk/Onboard-SDK-ROS/) package is used as the middleware on the SBC. The SBC also runs the outer-loop PID position controller and waypoint navigation algorithm. The state estimation and localisation of the UAV using fused GNSS and IMU sensor measurements is provided by the flight controllers. Both UAVs use a point to point wireless connection between the SBC and a laptop for remote monitoring and debugging. An Android mobile device connects to the DJI C1 controller to send commands to the on-board computer from a mobile application.

**Figure 2:**
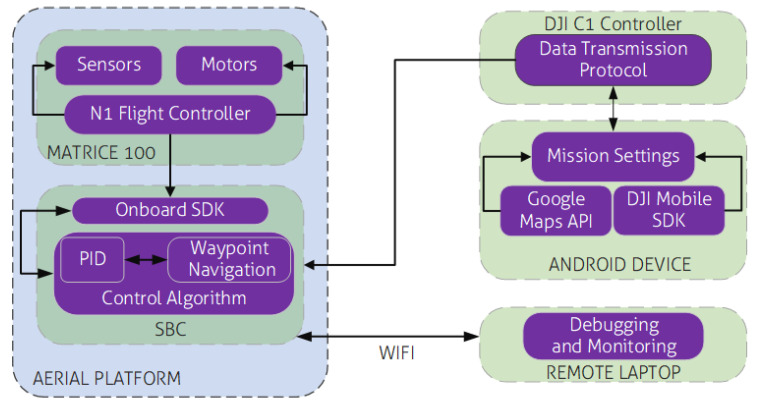
System Architecture of the UAV.

As shown in Figure 2, the SBC on the UAV is connected to a remote computer through a wireless hotspot created on the remote computer. This computer creates an SSH tunnel which gives access to the terminal on the aerial platform. This is used to monitor current mission status on the onboard computer. A high level mission planner was developed on Android as an app to plan waypoint missions on the UAV using the Google Maps API (https://developers.google.com/maps/documentation/android-sdk/intro) and DJI Mobile SDK (https://developer.dji.com/mobile-sdk/).

### 2.2. Underwater Acquisition Device

The hardware architecture of the underwater acoustic recorder is shown in Figure 4 and is made up of the following parts:

#### 2.2.1. Hydrophone

The hydrophone is the sensing element used to detect underwater sound. The hydrophone used in this article is a HS/150 omnidirectional hydrophone manufactured by Sonar Products Limited. The hydrophone has a resonant frequency of 150 kHz with receiving sensitivity of −204 dB re 1 V/μPa and a frequency range of 0.1–180 kHz.

#### 2.2.2. Preamplifier

The signals recorded by the hydrophone are amplified by a preamplifier which consists of an active band pass filter designed using the Texas Instruments Filter Design Tool [48]. As harbour porpoises are known to make narrow bandwidth high-frequency sounds between 115 and 180 kHz with a peak centre frequency of 135–140 kHz lasting about 44–113 μs [49,50], the amplifier was designed to use a 40 dB 2-stage 4th order Butterworth filter with a centre frequency of 130 kHz and a 60 kHz bandwidth with 3 dB cutoff points at 90 kHz and 150 kHz. The output of the preamplifier is then passed into a summing amplifier with a 1.65 V bias to prevent the signals from clipping at the unipolar input of the Analog to Digital Converter.

#### 2.2.3. Signal Processing Board

The signal from the summing amplifier is transmitted to a 12-bit single-ended ADC present on the STM32F7-Discovery development board. With a core clock of 216 MHz, the board serves as the central processing unit of the data acquisition system. It is used to process the hydrophone signals which are stored as WAV files on a Micro SD card. Using the ARM CMSIS DSP libraries (http://www.keil.com/pack/doc/CMSIS/DSP/html/index.html), a 1024 bin real fast Fourier transform of the incoming signals is also computed with the result of each FFT computation transmitted to the SBC for visualisation.

## 3. System Software Architecture

### 3.1. Firmware of the Data Acquisition Device

**Figure 3:**
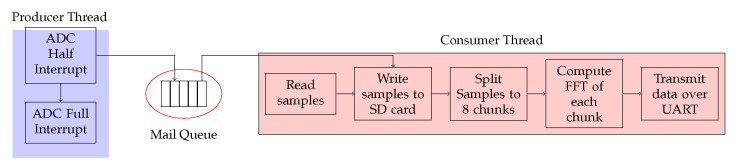
Software flowchart for the firmware present on the microcontroller.

The firmware on the data acquisition device enables the signals from the hydrophones to be processed and stored on a Micro SD Card. Written in C and leveraging the Hardware Abstraction Layer libraries (HAL) from ST Microelectronics, the firmware runs on a Real Time Operating System (FreeRTOS) [51]. The device can record uncompressed 16-bit WAV files at a sample rate of 400 kHz. The software flowchart is shown in Figure 3. A producer and consumer thread are created with the producer thread initiating the ADC conversion. The ADC sampling time is configured to start conversions on a trigger update from a timer. Using a circular Direct Memory Access (DMA) buffer, the ADC writes the converted hydrophone signals to a buffer which contains 16,384 samples or 32 kB of data. When half of the buffer is filled, an interrupt is triggered, and the buffer is placed onto a queue. The consumer thread then dequeues the data and writes to the SD Card. After writing, the 8192 samples are divided into eight windows and a real fast Fourier transform is applied on each window. The dominant frequency component in each window is calculated and filtered between 90 kHz and 150 kHz. The output of each iteration is finally transmitted over a serial UART Interface to the onboard computer for visualisation.

### 3.2. UAV Software Architecture

#### 3.2.1. Estimation

**Figure 4:**
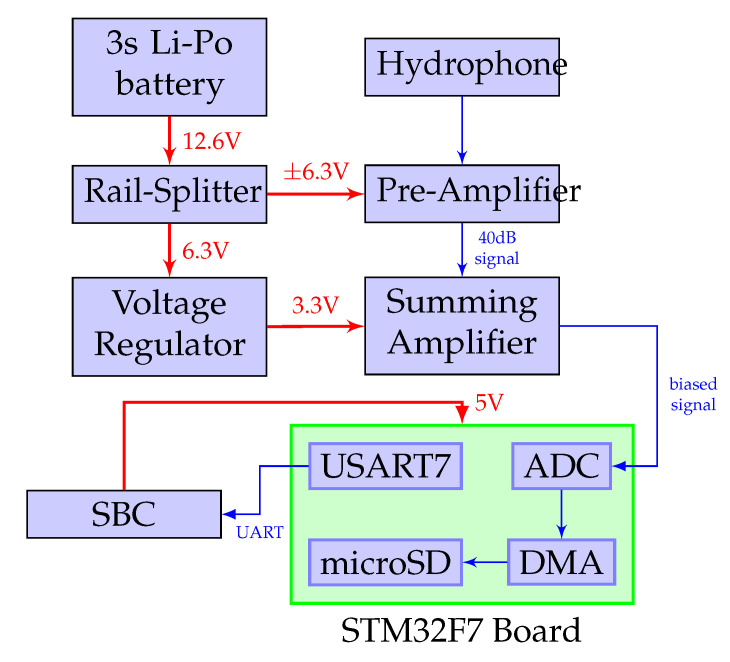
The block diagram of PorpDAQ. The preamplifier circuit is powered by a 3-cell Lithium-Polymer battery with a TL2426 “Rail-Splitter” creating a virtual ground between the rails. A 3.3 V voltage regulator is also used to provide power to the summing amplifier. The PorpDAQ is powered through USB and connected to the onboard computer.

Estimating the state of the UAV is required to apply a control signal. For mobile robots, a feedback of the motion of a robot and an estimate of its position in the environment is required for safe navigation. This information together with a knowledge of a robot’s speed is required in multirotor aerial vehicles to maintain in-air stabilisation. The sensors on a UAV usually provide **i:** inertial measurements from an Inertial Measurement Unit (IMU). The IMU provides the angular rates from the gyroscope and linear acceleration from the accelerometer. Measurements from both sensors are used to compute the attitude of the UAV. The **ii:** position measurements from GPS sensors to determine the absolute position of the UAV in the world frame. These measurements can be used with the sensors that produce relative position with respect to a reference point to improve the estimation of the UAV’s position. Relative position measurements are provided by computing the odometry of the UAV. This is usually through integrating the measurements of the IMU over a period of time. Additionally, there are visual methods of computing odometry available which allow you to estimate the change of the relative position of a UAV using cameras [52,53]. In this work, the fused IMU and GPS sensor measurements provided by the Onboard SDK are used to estimate the position of the UAV (Figure 4).

#### 3.2.2. UAV Control

A cascaded control strategy is adopted whereby an outer loop position and yaw controller is used with the attitude controller present in the flight controller hardware. The attitude controller which is based on a Proportional-Integral-Derivative (PID) controller provides the desired output to the motors on the UAV during flight [54]. The position and yaw controller also based on PID control are used to generate the desired velocities for the attitude controller.

The position controller is implemented to control the translational velocity of the UAV while the yaw controller is used to set the desired bearing towards the target position. The desired position is passed as a control input to the position controller while the desired yaw position serves as the input to the yaw controller. The desired velocities of the UAV is computed as:

(1) $$v=K_{p}\varepsilon v+K_{i}\int \varepsilon vdt+K_{d}\dot{\varepsilon v}$$

while the yaw is given as:

(2) $$\psi =K_{p_{\psi }}\delta \psi +K_{i_{\psi }}\int \delta \psi dt+K_{d_{\psi }}\dot{\delta \psi }$$

where $K_{p}$, $K_{i}$ and $K_{d}$ are constant proportion, integral and derivative gains for controller, *v* is the controller output for the velocity in the global frame and ψ is the controller output for the yaw angle of the UAV. εv is the error between the desired and current velocities on the xyz axis and δψ is the error between the target yaw angle and the current yaw angle of the UAV. The output of both controllers is passed into the attitude controller on the autopilot which sends the appropriate pulse width modulation signals to the motors. The measured position is fed back to the input of the position controller at 50 Hz. This process is repeated until the UAV reaches its desired position. For position control, $K_{p_{xyz}}$ = 1.95, $K_{i_{xyz}}$ = 0.001 and $K_{d_{xyz}}$ = 0.00005 while $K_{p_{\psi }}$ = 1.1, $K_{i_{\psi }}$ = 0 and $K_{d_{\psi }}$ = 0.05 for yaw control.

#### 3.2.3. High Level Mission Planner

The UAV mission planner was developed on the Android platform and written in Java. As mentioned earlier, map data is leveraged through the Google Maps SDK for Android and the DJI Mobile SDK was used to fetch telemetry data from the UAVs to display on the screen. The planner is compatible with the DJI M100 and the A3/N3 DJI flight controller. On the mission planner, a user is able to set mission parameters such as GPS coordinates (latitude and longitude), altitude for the UAV to fly at during the mission, the speed of the UAV, if the UAV is required to land and sample at a waypoint, the sampling time at each waypoint and an action for the UAV to compete at the end of the mission.

The data is transmitted to the onboard computer as a bytestream using the DJI Transparent Data Transmission Protocol (https://bit.ly/32STlhA). To activate this, the remote controller of the UAV needs to be connected to the planner through a USB connection. For the DJI N3 and A3 flight controllers, Lightbridge 2 (https://www.dji.com/uk/lightbridge-2) is required to transmit data from the planner to the UAV. The control algorithm on the onboard computer then decodes the data transmitted from the mobile device and converts to a usable format on the UAV. It also computes local position offsets from the global position estimates and uses these values to set velocity commands on the UAV. The data frame header is mapped to different functions which enable other UAV functionality such as taking off, landing, starting and aborting missions. A data parser is used to convert the data from the onboard computer for waypoint and mission configurations back into their respective data-types.

#### 3.2.4. Flight Control

**Figure 5:**
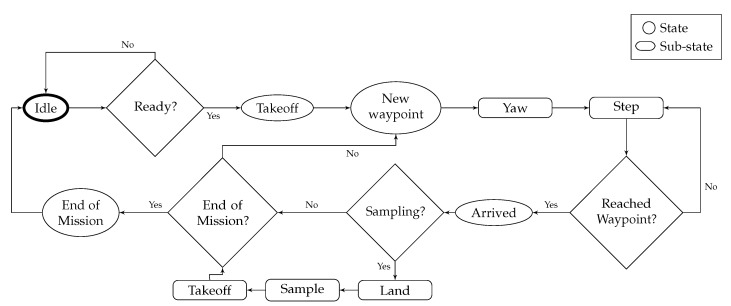
Software flowchart of the flight control algorithm on the on-board computer. Sub-states are actions that are triggered between 2 states to enable a transition to the next state. The UAV is in an **IDLE** state at the beginning of the mission.

The flowchart of the flight control algorithm is defined in Figure 5. The high level diagram describes the behaviour of the system once the user starts the mission from the mobile device.

The mission starts when the user presses the start button on the mobile device. At this stage, the UAV takes off to an altitude of 1 m and waits for an operator to visually confirm a successful take-off before transitioning into a **NEW WAYPOINT** state. In **NEW WAYPOINT**, the UAV uses the fused GPS measurements to estimate its global position and compute the distance to the next waypoint in a local frame using the following equations:

(3) $$\begin{array}{c} x_{a}=\left(lat_{a}-lat_{i}\right)R_{E}\\ y_{a}=\left(lon_{a}-lon_{i}\right)cos\left(lat_{a}\right)R_{E}\\ z_{a}=alt_{a}-alt_{i} \end{array}$$

where $R_{E}$ = 6,378,137 m is the Earth radius, $lat_{a}$, $lon_{a}$, $alt_{a}$ are the latitude, longitude (in degrees) and altitude (in metres) of the target waypoint respectively. The *i* subscript corresponds to the current position of the UAV.

Before navigating to the next location, the UAV calls the **YAW** action which sets the bearing of the UAV from its current position to the next waypoint using the following equation:

(4) $$\psi_{desired}=atan2\left(\frac{x_{d}-x_{c}}{y_{d}-y_{c}}\right)$$

where $\left[x_{d},y_{d}\right]$ are the desired *x* and *y* positions and $\left[x_{c},y_{c}\right]$ is the current position of the UAV. The output of this equation is passed into the position controller described in Section 3.2.2. Once the target yaw angle is set, the system continuously calls the **STEP** action. **STEP** sets the required velocity commands on the *z* axis. Once the UAV reaches its desired flight altitude, the lateral velocity commands are set for the *x* and *y* axis using the equation below.

(5) $$\vec{P_{des}}=v\left[\begin{array}{c} \frac{x_{d}}{\left\|\vec{P_{d}}\right\|}\\ \frac{y_{d}}{\left\|\vec{P_{d}}\right\|} \end{array}\right]$$

$P_{des}$ is a vector which contains the target *x* and *y* commands while $P_{d}$ is a vector containing $x_{d}$ and $y_{d}$. This enables the UAV to fly in straight line paths between waypoints.

Once the UAV reaches the waypoint, the system moves to the **ARRIVED** state. If there’s sampling required at the waypoint, the UAV attempts to land. Once the UAV successfully lands, the motors turn off and the onboard computer sends a signal to the data acquisition device to start sampling for a user defined period as set on the mobile app. This approach to landing and sampling reduces energy consumption which increases battery life for longer mission times and also reduces the impact of UAV noise on the environment. After sampling, the take-off action is called and the onboard computer sends another signal to the data acquisition device to stop sampling. If there are no waypoints left, the UAV enters a **FINISHED** state, otherwise, it goes back to a **NEW WAYPOINT** state. This state transition also applies if the UAV is not performing a task. At the **FINISHED** state, the UAV completes a hover, land or fly home action. The hover action enables the UAV to hover at the final waypoint, while the **fly home** function flies the UAV to the original take-off position. The **fly home** function provided by the manufacturer was overridden as the default Return-To-Home function on the UAV is defined as the last take-off position which is not suitable for missions that require persistent landing and take-off. The **fly home** implementation records the GPS position from the first take-off point and computes the Cartesian distance using Equation (3) before flying to that location.

## 4. Results

The performance of the systems was validated by performing a combination of hardware experiments and simulations. The performance of the data acquisition device was tested in an indoor tank (Figure 9) at Loughborough University while autonomous flights on the quadcopter were tested outdoors. The integration of both systems was tested with a combination of hardware in the loop simulations and water experiments in a tank.

### 4.1. Outdoor Experiments

**Figure 6:**
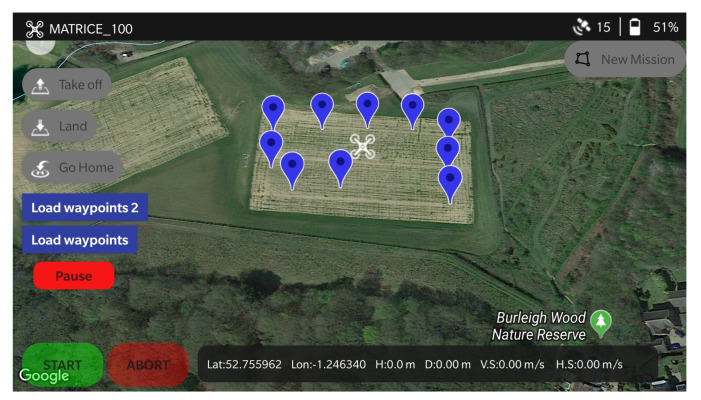
Interface for the high level mission planner.

The navigation algorithm of the system was tested outdoors at the Holywell football fields at Loughborough University (Lat: 52.755964, Lon: −1.246318). The M100 described in Section 2.1 was configured to run multiple missions, some of which involved persistent landing at 10 waypoints. The UAV was configured to “sample” at those waypoints for 10 s. Since the experiments were not being run over water, the data acquisition system was not attached. The UAV landed at the waypoints and took off to the next waypoint after 10 s. These missions were tested at a horizontal and vertical speed of 2 m/s with a constant relative altitude of 5 m from the UAV’s take-off position as shown in Figure 6. During the tests, the M100 was unable to complete the preconfigured 10 waypoint mission at 1m/s as it kept running out of battery before the end of the mission.

**Figure 7:**
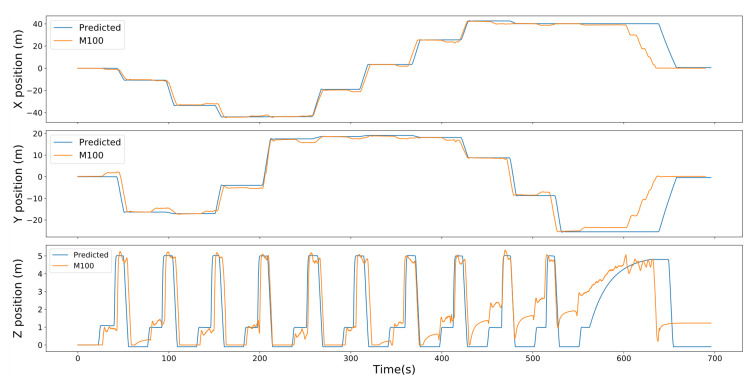
Trajectory of the the M100 compared against the desired trajectory in the *x*, *y* and *z* axis.

Figure 7 compares the trajectory of the M100 against a desired trajectory of one of the missions flown. At 2 m/s, the M100 completed the mission in 11:49 against a predicted time of 11:22. A Pearson correlation coefficient of each axis for both UAVs was calculated to compare the relationship between the predicted and actual trajectories on each axis.

**Figure 8:**
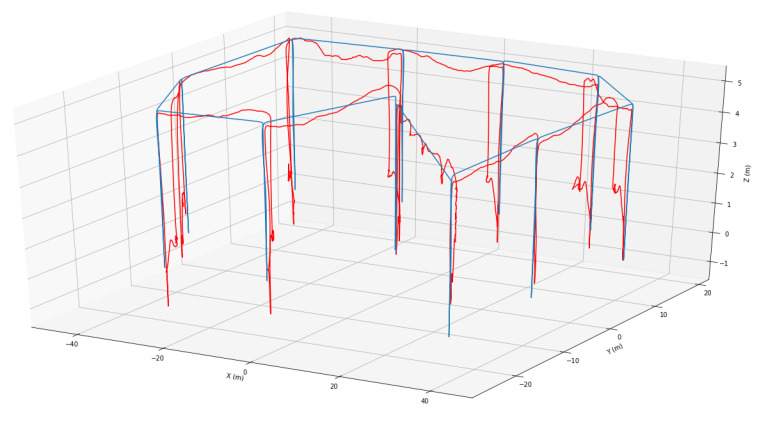
3D Trajectory of the M100 compared against a reference trajectory.

The results show there is a strong correlation between the predicted and actual trajectories between both missions with a correlation coefficient $r_{x}$ = 0.943, $r_{y}$ = 0.856 and $r_{z}$ = 0.793. The actual trajectory of the M100 during the missions closely match the reference trajectories and the UAV reaches the desired altitude at each waypoint as shown in Figure 8. During the last three waypoints, the UAV drifted on the vertical axes while it yawed towards the next waypoint instead of hovering at 1 m; however, it still flew to the desired altitude and was able to arrive at the desired waypoints. Additionally, it is also important to note the behaviour of the M100 on the *z* axes from t=550 in Figure 7. At this point, the UAV has completed the mission and is configured to fly back “home” by maintaining a constant altitude within ±20 cm of the desired *z* position. Currently, the M100 only uses a barometer to estimate altitude which makes it subject to infrequent drifts in its vertical position. The algorithm reacts to this and sends a command to UAV to adjust its vertical position till it reaches the setpoint. An adverse effect of this is on windy days; the UAV wastes a lot of time trying to maintain an altitude at the desired position which leads to increased battery consumption and longer flight times. However, some of these issues can be mitigated by increasing the tolerances and adjusting the sensitivity of the integral action on the controller.

### 4.2. Underwater Acoustics Device

The flight navigation and underwater recording capability was tested by simulating multiple missions at the same location in the DJI simulator with the N3 quadcopter. The data acquisition device starts recording once the UAV lands at each waypoint and recording is stopped once it takes off. As set in the outdoor missions, the data acquisition device was programmed to listen for 10 s and write the data to onboard storage. The onboard computer also runs another ROS node which is used to display the FFT output computed by the data acquisition device using the OpenCV library [55].

**Figure 9:**
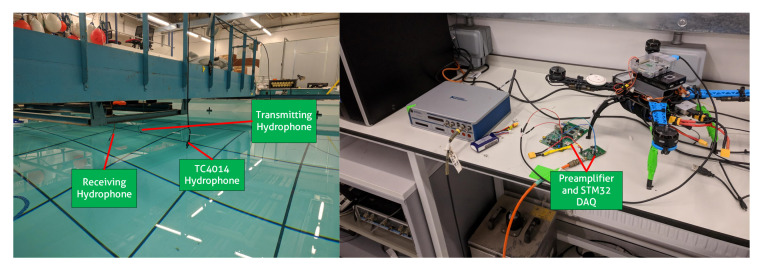
Indoor tank setup of the source transducer, PorpDAQ and reference system.

The data acquisition device was tested in a tank measuring 9.5 m × 5.5 m × 2 m. An analysis of the system was conducted with known frequencies transmitted across the tank and received by the data acquisition device and a reference system as shown in Figure 9. All hydrophones were deployed at a depth of 1 m. A simulated harbour porpoise signal lasting 100 μs with a peak centre frequency of 130 kHz and an interclick duration of 100 ms was then generated in MATLAB and continuously transmitted through a National Instruments VirtualBench (https://www.ni.com/en-gb/shop/select/virtualbench-all-in-one-instrument). The VirtualBench was set to a sampling frequency of 1 MHz and a HS/150 hydrophone was connected to act as a transmitter. The reference system is made up of a Teleydne Reson TC4014 Hydrophone and a Teledyne A2002 Amplifier set with a bandpass frequency of 100–200 kHz. The amplifier was connected to a NI-6363 DAQ with a sample rate of 400 kHz. A breakdown of both system’s specifications is shown in Table 1.

Where possible, the reference system was set to match the data acquisition device. The source level of the signal SL was calculated using $SL=TVR+20log_{10}\left(V_{out}\right)$ where TVR is the transmit voltage response of the transmitting hydrophone and $V_{out}$ is the output voltage from the VirtualBench. The given specification of the hydrophone’s TVR is 145 dB re 1 μPa/V at 150 kHz. With a maximum output voltage of 24 V from the VirtualBench, the theoretical source level is given as 172 dB re 1 μPa${}_{\mathrm{pp}}$. However, this value is expected to be lower as the transmitting signal is below the resonant frequency of the hydrophone and a power amplifier is not being used to drive the signal. By taking multiple measurements at different distances and different voltages, the actual source level of the signal is calculated using $SPL_{rms}=SL-TL$ where $SPL_{rms}$ is the root mean square sound pressure level in dB re 1 μPa and TL is the transmission loss. By recording the SPL using the reference hydrophone at 1 m and given that the transmission loss can be calculated using $TL=20log_{10}\left(R\right)+\alpha R$ (assuming spherical spreading) where *R* is the distance between the receiver and the transmitter in metres and α is the absorption coefficient at the centre frequency of the transmitted signal. The absorption coefficient is assumed to be negligible as the receiver is very close to the source, therefore $TL=20log_{10}\left(R\right)$. The actual source level is calculated to be 150 dB re 1 μPa${}_{\mathrm{rms}}$ which is about 15 dB lower than reported source levels for wild porpoises and within the source levels for captive porpoises [56].

**Figure 10:**
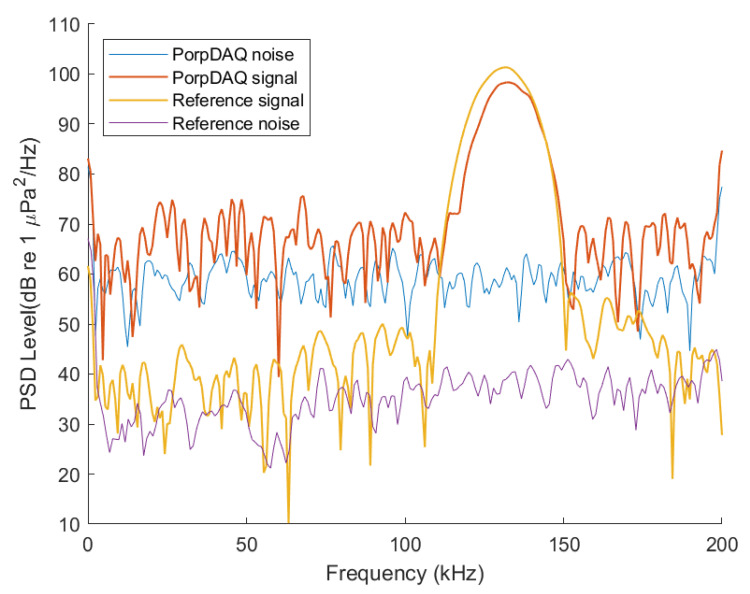
Power spectral density of the recorded background noise from the PorpDAQ (blue line) and the background noise from the reference system (purple line). The power spectral density of a click captured by both systems are also shown.

**Figure 11:**
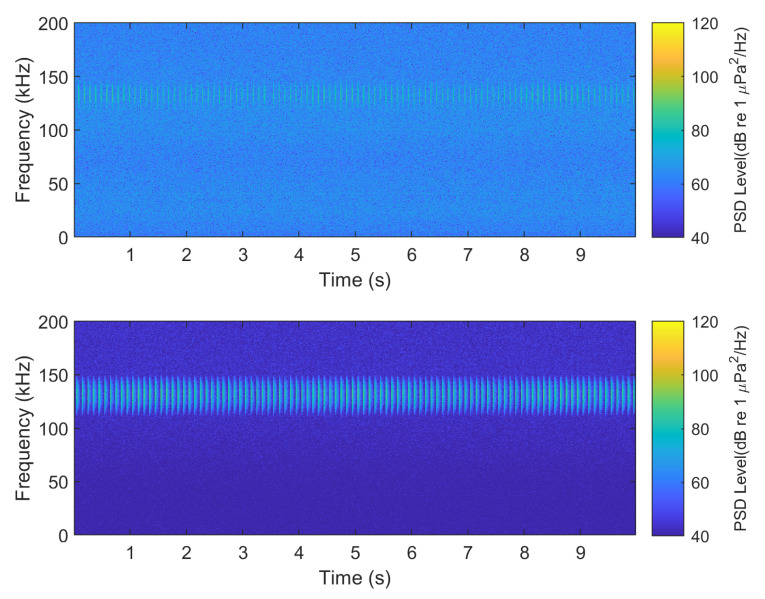
Spectrogram of the PorpDAQ (**top**) and the reference system (**bottom**) of a simulated click recorded by both systems.

**Figure 12:**
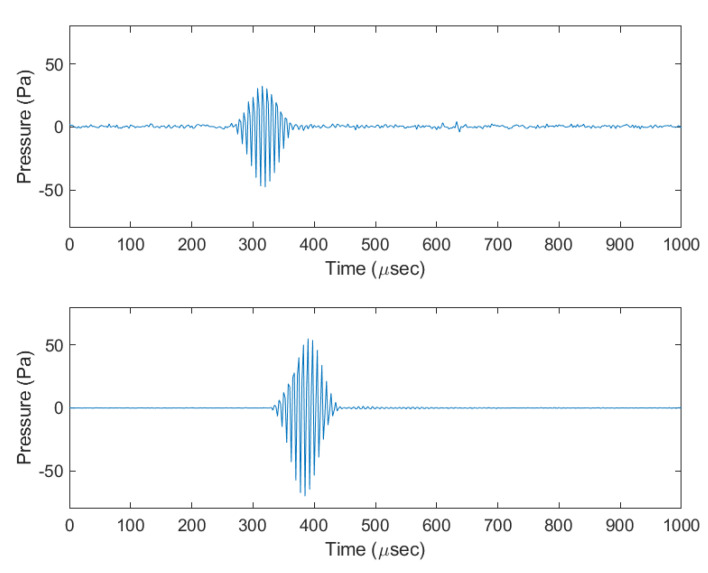
Time domain signal of a single click recorder by the PorpDAQ (**top**) and the reference system (**bottom**). The waveform shown at the top has a recorded peak frequency of 131.8 kHz while the bottom waveform is recorded at 129.7 kHz.

The power spectral density (PSD) of the clicks from both systems (Figure 10) was computed using Welch’s PSD algorithm with a 300 point segment length and a 50% overlap. As shown in Figure 11, the spectrogram shows an example WAV file from both devices capturing data for 10 s. Within this window, there are 98 pulses present for the data acquisition recorder while the reference system records all 100 pulses. Figure 12 shows an example waveform from both devices.

The average centre frequency of the click for the data acquisition device was at 131.6 kHz with an amplitude of 95.7 dB re 1 μPa${}^{2}$/Hz while the centre frequency on the reference system was 130.4 kHz with an amplitude of 96.9 dB re 1 μPa${}^{2}$/Hz. The average centre frequency, amplitude and $SPL_{\mathrm{rms}}$ recorded on the data acquisition device and the reference system together with the difference between the average SPL of both systems is shown in Table 2. The SPL for both systems is quite low compared to the expected levels from harbour porpoises but as mentioned earlier, this is expected due to the limitations of the maximum output of the testing equipment.

**Figure 13:**
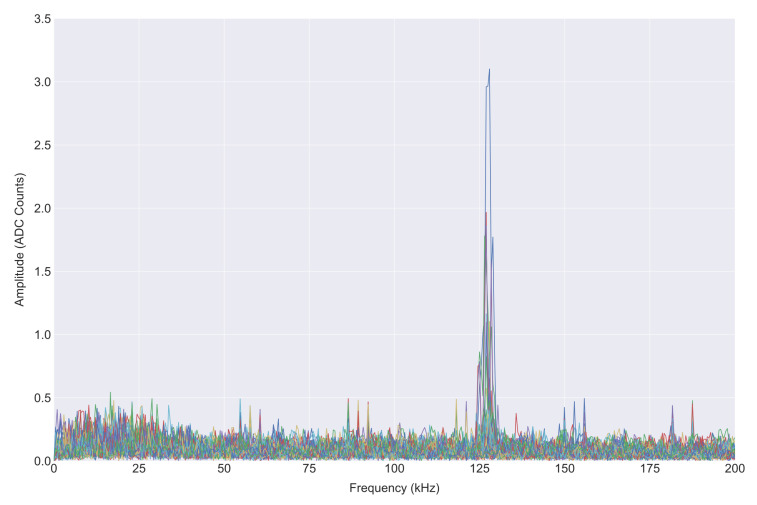
Real-time ADC frequency component on the PorpDAQ for a 10-s recording.

**Figure 14:**
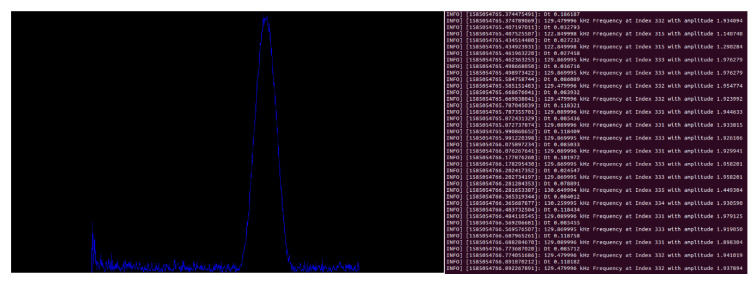
Unscaled FFT output displayed on an image stream in real-time using OpenCV and the dominant frequency in each computation printed to a terminal.

The results of the real-time fast Fourier transform from the data acquisition device on one of the recordings made during a mission is shown in Figure 13. The result of each FFT operation with a dominant frequency between 90 and 150 kHz is transmitted at 115.2 Kb/s and displayed to an operator as a simple image in real-time as shown in Figure 14. The dominant frequency in each bin is also printed to a terminal. Filtering the output for frequencies of interest reduces the amount of data needed to transmit over the serial interface and enables the system to log all occurrences of the frequency of interest on the onboard computer.

One potential issue to consider is how the introduction of noise and visual stimuli to the environment by UAVs affect the physiology and behaviour of marine mammals in areas where the system is deployed. There are currently no data on the responses of harbour porpoises to the presence of UAVs, but it is possible to make assumptions on behaviour based on data available for other marine mammals. The main disturbances from UAVs come from noise and visual cues from the shadow of the UAV [57]. There have been studies conducted to show the impact of UAVs on marine mammals with results ranging from animals showing signs of distress to animals exhibiting no discernible reaction. For example, while evaluating the use of aerial imagery using multirotor UAVs in seal breeding colonies, the reaction of animals to the presence of UAVs varied between the type of UAV flown [58]. Breeding and moulting grey seals showed more alert behaviour especially at flight altitudes below 30 m which in some cases caused the seals returning to sea. It was also observed that both groups of harbour and grey seals reacted differently to multirotor UAV noise based on age and sex; however, the noise levels of the UAVs used in this experiments were not quantified. Consumer UAVs were flown to evaluate the behavioural responses of bottlenose dolphins and Antillean manatees [59]. Dolphins exhibited low responsiveness to UAV flights and in general only responded to a small proportion of the observations, but there is an increasing amount of evidence to suggest the presence of UAVs at altitudes less than 30 m elicits a response from bottlenose dolphins [60]. The manatees, on the other hand, exhibited a sensitivity to the movements of the aircraft and displayed evasive behaviour towards the UAVs at altitudes as high as 100 m.

Noise levels of two types of multirotor UAVs (DJI Inspire 1 and SwellPro SplashDrone) were tested and the noise profiles of both UAVs in-air and underwater were recorded [61]. The fundamental frequency of the Inspire drone was recorded at 150 Hz with most of the energy present at 450 Hz. The mean source level was recorded at 101 dB re 1 μPa${}_{\mathrm{rms}}$. A similar result for this UAV was also observed in Erbe and Parsons et al [62] albeit with slightly lower received levels. The SplashDrone was recorded with a fundamental frequency of 60 Hz with peak received levels at 200 Hz and a mean source level of 95 dB re 1 μPa${}_{\mathrm{rms}}$. Overall, UAV noise is seen to be only quantifiable underwater when UAVs are flown at 5 m or 10 m above the water surface and the energy from in-air UAVs does not transmit into the water surface. Results from other UAVs recorded in [62] shows broadband noise with distinct tones of 80 Hz and 160 Hz. The authors also concluded that the maximum received levels underwater from multirotor UAVs are lower than commercial and military aircraft flying at altitudes greater than 400 ft but higher than noise recorded from small fixed wing aircraft.

In general, flight altitude is seen to be an important factor in terms of noise emitted from UAVs. However, there seems to be no discernible difference between disturbance from noise or disturbance from UAV shadows with different species of marine mammals reacting differently. Research suggests ambient noise in the environment likely masks the noise emitted by UAVs in certain habitats; however, noise levels are higher near the water surface and in shallow waters [61,62]. Recommendations and best practices for the usage of UAVs in marine mammal research suggest flying UAVs at highest possible altitudes to gain usable data, minimising UAV movements and approach angle and also taking into consideration if the noise from the UAV falls within the frequency range of the species’ hearing [59,60]. It is important to note that the lack of an observed behavioural response does not necessarily suggest a lack of impact. Therefore, there are also other steps to be taken in order to further minimise impact. One suggestion is to consider the effects of the type of UAV on specific marine mammal species, i.e., assessing if the UAV’s airframe matches the profile of potential predators of the marine mammal species.

The dominant source of noise from UAVs originates from the propulsion system [63] and given the profile of the UAVs used in this article is similar to the ones measured in [61,62], i.e., all UAVs share the same model of motors, ESCs and propellers, it is assumed they have the same noise profile. When compared to the audiogram of odontocetes and delphinidae presented in [64,65], it is unlikely harbour porpoises will be able to hear the noise emitted from the class of multirotors used in this article. Therefore, after considering other noise mitigation factors taken in this study such as the motors turning off after the UAV has landed, the potential noise impact of UAVs on marine mammals is expected to be low.

## 5. Conclusions

In this article, a method for recording harbour porpoise vocalisations using an unmanned aerial vehicle was successfully demonstrated. The system described enables the autonomous navigation of UAVs in marine environments and the autonomous recording of marine mammal vocalisations. Using relatively low-cost hardware, the potential applications of UAVs in passive acoustic monitoring is described. Additionally, the use of easily accessible open source software has enabled the development of a system that can be easily adapted for other applications. The outdoor flight results demonstrate the feasibility of persistent autonomous landing and navigation of a UAV while the indoor experiments and simulations demonstrate the use of a lightweight, low-cost data acquisition system that can be triggered to record automatically once the UAV lands at a target area. The autonomous flight navigation software is compatible with a range of DJI flight controllers and can be easily deployed and modified on any system running the ROS framework. The control parameters used for the UAVs in this article during the outdoor tests were not extensively tuned and some of the issues regarding the UAVs’ robustness to external disturbances when flying can also be solved by tuning the PID parameters.

While the acoustic recorder has been designed to record sound within a specific frequency band for harbour porpoises, the modular design of the system allows for an extension of the system to record other species vocalisations. As the firmware on the PorpDAQ only processes incoming data from the ADC which is connected to a preamplifier, a broadband preamplifier can be designed and connected to the PorpDAQ to enable the processing of species vocalisation across all frequencies of interest up to a sampling rate of 400 kHz. The inclusion of a form of real-time spectral analysis also enhances the usability of the system and creates a platform that can be extended to implement real-time species detection on low-cost hardware. There are some drawbacks to the use of low-cost hardware such as the low dynamic range of the microcontroller and the high noise floor of the ADC compared to the reference system. A low noise ADC can be used to interface with the microcontroller through the SPI bus but this method increases the complexity of the system in terms of implementation. Other hardware constraints on the system which is generally found in low cost microcontrollers is the lack of enough onboard memory. The development board used in this article has about 320 kB of RAM present which is relatively generous but still limited when a high sampling rate is required. About half of the memory present is reserved for the queue to act as a buffer while the data is being written to the SD card. The write speed on the SD card after tweaking the performance of the system was around 20 Mb/s, which creates a bottleneck in the system. It would be possible to achieve a higher sampling rate if either more memory is available to increase the size of the buffer or the speed of each FFT computation is improved. The former is achievable by adding external memory such as static RAM through the flexible memory controller present on the microcontroller while the latter would require a different architecture as the FFT computation time for floating point numbers on the recorder scales linearly regardless of the number of bins. One option would be to adopt the improved M7 architecture based on the STM32H7 microcontroller which has a core clock of 480 MHz and a 208 MHz SDMMC interface which theoretically quadruples the write speed on the current system. Other options include FPGA-based systems, some of which have been implemented in underwater acoustic localisation systems [66]. However, despite these limitations, the custom data recorder produces good performance at a much lower cost compared to other options available.

Moving forward, there are active plans to improve the real-time spectral analysis pipeline and provide a graphical user interface to display the information to an observer. There are active developments in deploying the system in a waterproof airframe and conduct outdoor tests in marine environments to further validate the work described in this article. Additionally, there is also ongoing research on the deployment of a multi-UAV system for recording underwater acoustic data from multiple locations simultaneously to increase coverage and reduce mission times.

## Figures and Tables

**Table 1:** Specification of both the data acquisition system and the reference system.

Specification	PorpDAQ	Reference System
ADC Resolution	12-bit	16-bit
Sampling Rate	400 kHz	400 kHz
Output Range	3.3 V	±10 V
Hydrophone Sensitivity	−204 dB re 1 V/ μPa	−180 dB re 1 V/μPa
Amplifier Gain	36.5 dB	20 dB
Combined Sensitivity	−167.5 dB re 1 V/ μPa	−160 dB re 1 V/ μPa
Dynamic Range	72 dB	96 dB

**Table 2:** Performance comparison between the reference system and the data acquisition device.

Device	Recorded ${SPL}_{\mathrm{rms}}$	Expected ${SPL}_{\mathrm{rms}}$	Recorded ${SPL}_{\mathrm{pp}}$	Expected ${SPL}_{\mathrm{pp}}$	Average Centre Frequency (kHz)
PorpDAQ	147.6	150	159.1	160	131.6
Reference System	149.7	150	160.9	160	130.4
